# An Evidence-Based Antimicrobial Stewardship Smartphone App for Hospital Outpatients: Survey-based Needs Assessment Among Patients

**DOI:** 10.2196/mhealth.5243

**Published:** 2016-07-06

**Authors:** Christianne Micallef, Monsey McLeod, Enrique Castro-Sánchez, Myriam Gharbi, Esmita Charani, Luke SP Moore, Mark Gilchrist, Fran Husson, Ceire Costelloe, Alison H Holmes

**Affiliations:** ^1^ National Institute for Health Research (NIHR) Health Protection Research Unit in Healthcare Associated Infection and Antimicrobial Resistance Imperial College London United Kingdom; ^2^ Addenbrooke`s Hospital Pharmacy Department Cambridge University Hospitals National Health Service (NHS) Foundation Trust Cambridge United Kingdom; ^3^ School of Pharmacy Centre for Medication Safety and Service Quality University College London London United Kingdom; ^4^ Imperial College Healthcare National Health Service (NHS) Trust Pharmacy Department London United Kingdom; ^5^ Imperial College Healthcare National Health Service (NHS) Trust Infectious Diseases Department London United Kingdom

**Keywords:** mobile electronic devices, mHealth, mobile health, antimicrobial resistance, patient involvement

## Abstract

**Background:**

Current advances in modern technology have enabled the development and utilization of electronic medical software apps for both mobile and desktop computing devices. A range of apps on a large variety of clinical conditions for patients and the public are available, but very few target antimicrobials or infections.

**Objective:**

We sought to explore the use of different antimicrobial information resources with a focus on electronic platforms, including apps for portable devices, by outpatients at two large, geographically distinct National Health Service (NHS) teaching hospital trusts in England. We wanted to determine whether there is demand for an evidence-based app for patients, to garner their perceptions around infections/antimicrobial prescribing, and to describe patients’ experiences of their interactions with health care professionals in relation to this topic.

**Methods:**

A cross-sectional survey design was used to investigate aspects of antimicrobial prescribing and electronic devices experienced by patients at four hospitals in London and a teaching hospital in the East of England.

**Results:**

A total of 99 surveys were completed and analyzed. A total of 82% (80/98) of respondents had recently been prescribed antimicrobials; 87% (85/98) of respondents were prescribed an antimicrobial by a hospital doctor or through their general practitioner (GP) in primary care. Respondents wanted information on the etiology (42/65, 65%) and prevention and/or management (32/65, 49%) of their infections, with the infections reported being upper and lower respiratory tract, urinary tract, oral, and skin and soft tissue infections. All patients (92/92, 100%) desired specific information on the antimicrobial prescribed. Approximately half (52/95, 55%) stated it was “fine” for doctors to use a mobile phone/tablet computer during the consultation while 13% (12/95) did not support the idea of doctors accessing health care information in this way. Although only 30% (27/89) of respondents reported on the use of health care apps, 95% (81/85) offered information regarding aspects of antimicrobials or infections that could be provided through a tailored app for patients. Analysis of the comments revealed the following main global themes: knowledge, technology, and patient experience.

**Conclusions:**

The majority of respondents in our study wanted to have specific etiological and/or infection management advice. All required antimicrobial-related information. Also, most supported the use of electronic resources of information, including apps, by their doctors. While a minority of people currently use health apps, many feel that apps could be used to provide additional support/information related to infections and appropriate use of antimicrobials. In addition, we found that there is a need for health care professionals to engage with patients and help address common misconceptions around the generation of antimicrobial resistance.

## Introduction

The increasing burden of antimicrobial resistance is a well-known global phenomenon which requires national [1] and international collaboration [2] for effective prevention, control, and management. Indeed, it is estimated that antimicrobial resistance (AMR) currently claims 50,000 lives per year across Europe and the United States [3]. The current forecast suggests that if the situation remains unchecked, AMR will be the leading cause of death in humans by 2050, surpassing cancer and diabetes-related diseases, claiming 10 million deaths annually and leading to economic losses of US $100.2 trillion [3].

Medical health technology apps offer a practical way to increase accessibility and navigability of medical information during routine provision of patient care by health care professionals. The benefits of medical apps to facilitate optimal patient care and self-management has recently been reported in sexual health [4], HIV/AIDS management [5], increasing fitness levels [6], and cancer prevention [7] in rural communities.

A number of studies have also explored and evaluated the use of apps targeted at people living with a range of clinical conditions, including diabetes [8], asthma [9], and stroke [10]. Apps have also been used to aid in self-care in women`s health [11] and mental health [12,13], and to support patients undergoing bariatric [14] and colorectal surgery [15]. These and other studies [16] delineate a consistent lack of governance around medical apps, thereby presenting significant implications for patient safety. Furthermore, many lack appropriate health care professional input and it is unclear how the needs of the target users were incorporated into the functionality and content of such apps.

A range of infection prevention and control and antimicrobial-related apps are available for medical and other clinical staff [17-20], however, to our knowledge there is as yet no study exploring the usability of apps for patients specifically targeting infections and/or antimicrobial use. In addition, a UK Wellcome Trust report [21] has provided important insights into the perception of antimicrobial resistance by the general public in the United Kingdom; the report concluded that there is a problem with communicating issues around antimicrobial resistance and an urgent need to educate the public in this field. Moreover, a recent systematic review [22] on the public’s knowledge and beliefs about antibiotic resistance confirmed the lack of understanding about the development of AMR and misperceptions about its causes. Providing patients and the public with appropriate information around infections and antimicrobial therapy using electronic resources, such as mobile phones, tablets, and personal computers, may help address this need.

We sought to explore the use of different antimicrobial therapy information resources with a focus on electronic platforms, including apps for portable devices by health care users at two large, geographically distinct teaching hospitals in England. We wanted to determine whether there is currently a need for an evidence-based app for patients, to garner public perceptions around infections/antimicrobial prescribing, and to describe patients’ experiences of their interactions with health care professionals in relation to this topic.

## Methods

### Overview

A cross-sectional study was designed using a 12-item survey investigating aspects of antimicrobial prescribing and electronic devices experienced by patients or their carers. The following participants were involved in the design of the questions and survey format: an infectious diseases physician; three hospital pharmacists, all with infection expertise; an academic lead nurse; a public health specialist; and a patient representative. A single open question invited respondents to annotate any comments they wished to make about the survey itself and/or any aspect on antimicrobial prescribing, resistance, and infections. Feedback from a patient hospital coordinator was used to check the survey compliance with National Health Service (NHS) readability requirements for materials provided to patients and the general public [23].The survey was then piloted with potential respondents for face validity.

### Data Exclusion

Inclusion criteria for participation included being ≥18 years of age and participants or their dependants having taken a course of antimicrobials in the previous 6 months. Any respondents who did not meet the above criteria were automatically excluded. Although requested, this study did not require ethics committee approval, as confirmed by the NHS Research Ethics Committee, as this work was considered to be a service evaluation.

### Recruitment

The survey was distributed by pharmacy outpatient staff to people attending four different hospitals across two acute NHS institutions, from September 15-30, 2014, in London and from November 18, 2014, to February 23, 2015, in Cambridge, until a convenience sample of 100 consecutive respondents had been reached. A master copy of the survey is provided in Multimedia Appendix 1. A raffle incentive—£30 shopping vouchers, one for each geographical location—was offered to respondents who completed the survey.

### Data Analysis

Data were anonymized and reported using descriptive statistics. Additionally, analysis of the free-text comments portion of the survey and the feedback provided by the respondents was conducted [24]. This was done by a single researcher (CM) who grouped the comments received into main and global themes. The themes were then reviewed and corroborated by the study team.

## Results

### Demographics and Ownership of Electronic Devices

A total of 100 surveys were returned, but only 99 (99.0%) surveys were eligible to be included in the analysis—one survey was excluded as the respondent was under 18 years of age. Not all respondents completed each question in the survey and this was taken into account in the analysis. For each question the number of respondents is given.

The median age of respondents (n=85) was 40 years (range 19-99), and 8% were over 70 years of age. Table 1 provides details on respondent demographics.

**Table 1:** Demographics of the patient sample .

Patient characteristics		n (%)
**Gender (female) (n=96)**		46 (48)
**Level of education (n=88)**		
	Degree	46 (52)
	Other qualifications	29 (33)
	No qualifications	13 (15)
**Personal electronic device owned (n=89)**		
	Desktop/laptop	62 (70)
	Android mobile phone	36 (40)
	Android tablet	15 (17)
	Apple mobile phone	40 (45)
	Apple tablet	27 (30)
	Windows-based mobile phone	6 (7)
	Windows-based tablet	3 (3)
	Blackberry mobile phone	1 (1)
	Other device	4 (4)
	No device	8 (9)

### Antimicrobial Prescribing and Obtaining Information on Antimicrobials/Infections

In all, 82% (80/98) of respondents had recently been prescribed antimicrobials for themselves and 19% (19/98) stated that their dependants/children were prescribed antimicrobials. A total of 87% (85/98) of respondents were prescribed an antimicrobial by a doctor, either in hospital or in the community. The remaining 13 individuals out of 98 (13%) stated that they acquired their prescription from the one of the following: dentist (4/98, 4%), nurse (5/98, 5%), hospital pharmacist (1/98, 1%), walk-in clinic while on holiday (1/98, 1%), and by self-prescription (2/98, 2%). When asked how they obtained information about the antimicrobials prescribed or the infection treated, 80% (74/93) said they had asked a health care professional (HCP) such as a doctor, pharmacist, or nurse. A total of 7% (6/93) had asked family and friends and 17% (16/93) had also searched the Internet. However, only 60% (51/85) of respondents were completely satisfied with the information they had been given by their HCPs.

In terms of the information that respondents wanted to know about their particular infection, 65% (42/65) wanted information on the etiology of the infection. Most common infections mentioned were as follows: upper and lower respiratory tract infections; urinary tract infections; and oral, skin, and soft tissue infections. In addition, 49% (32/65) wanted information on the prevention and/or management of their infection. When asked whether they wanted any specific information relating to the antibiotics prescribed, all the respondents who answered this question (92/92, 100%) desired at least one particular piece of information among the following: best time of day to take antibiotics, whether the antibiotics should be taken with food, whether alcohol could be consumed, whether the antibiotics could be taken at the same time as other medicines, what to do about a missed dose, and side effects. Some patients also added their own comments, for example, the patient information leaflet used, the ability of antibiotics to induce thrush, age group of people who could take the antibiotic, safety in pregnancy, and safety in blood disorders (eg, thalassemia).

Table 1 also provides information on the electronic devices owned by patients. While 30% (27/89) of patients used health care apps, 95% (81/85) completed a preference list of statements on aspects of antimicrobials and/or infections that could potentially be provided through a dedicated app for patients (see Table 2).

**Table 2:** Preferences for an antimicrobial app and patients' perceptions on the utilization of electronic platforms by health care professionals.

Parameter		n (%)
**Characteristics of a dedicated antimicrobial app for patients/public (n=85)**		
	Treatment of symptoms	64 (75)
	Side-effects of antimicrobials	60 (71)
	Duration of common infections	53 (62)
	Tips on how to reduce risk of getting common infections	49 (58)
	Are antimicrobials indicated for my infection?	48 (56)
	When do I need to see my doctor?	47 (55)
	Signs of bacterial versus viral chest infections	43 (51)
	Recording of antimicrobial treatment information	30 (35)
	Other information (free text)	5 (6)
**Patients’ perceptions on use of mobile electronic devices during a consultation with a doctor (n=95)**		
	Fine to use	52 (55)
	Not fine, they should not be using it	12 (13)
	Depends on the situation	22 (23)
	Other: specific scenario given	9 (9)

### Analysis of Respondents' Feedback

Additional insights on their experience with antimicrobials, infections, and apps were provided by 15% (15/99) of respondents, and are presented in detail in Table 3.

The main themes were patient information leaflets, information on infections/antimicrobials, technology awareness, and personal experience on infections/antimicrobial prescribing.

**Table 3:** Themes emerging from respondents’ feedback^a^.

Global theme	Main theme	Verbatim comment	Respondent characteristics
Knowledge			
	Patient information leaflets		
		*"Leaflets included with medicines are a pain. Too clumsy and contain information overload.”*	Male, no age supplied
		*“Leaflet is fine for most people to read but needs to be concise and to the point, for example, if you miss a tablet don`t take it. Tips: major things in BOLD writing.”*	Female, 60 years old
	Information on infections and/or antimicrobials		
		*“Would be useful to know why certain antibiotics are prescribed over others. In my case was prescribed antibiotics that didn`t work.”*	Female, 27 years old
		*“Length of time till symptoms don`t ease—implications of this.”*	Male, 29 years old
		*“Information needed on gaps one should leave between antibiotic courses, in order to reduce problems with resistance.”*	Male, 60 years old
		“Gap one should leave between antibiotic courses in order to decrease resistance.”	Male, 66 years old
		*“This survey is very interesting. It is making you actually think about the issues around taking medications.”*	Female, 48 years old
		*“Information is good but does not beat real medical assessment.”*	Male, 39 years old
Technology			
	Technology awareness		
		*“Prefer than app, comprehensive website optimized for phones and tablets.”*	Male, 33 years old
		*“This survey could be an APP!”*	Female, 43 years old
		*“Doctors need to keep up to date with technology.”*	Male, 40 years old
Patient experience			
	Personal experience with infections/antimicrobial prescribing		
		*“I had severe allergic reaction to several antibiotics following brain operation and now being seen by [a specialist].”*	Female, 57 years old
		*“Antibiotics given too often to patients and GPs [general practitioners] see it as a quick fix to get rid of the patients. There should be limitations in place to decrease antibiotic prescribing.”*	Female, 24 years old
		*“Child became ill again but GP would not issue another dose, suggested other methods and asked to return if became worse.”*	Female, 57 years old
		*“Antibiotics are good but immune system becomes so depleted after a while. When younger, had tonsillitis and always on antibiotics and effects being experienced in adulthood.”*	Female, 32 years old

^a^Only superfluous comments were excluded (eg, “great” or “I have a carer”); the rest are all included in the table.

## Discussion

### Principal Findings

In this study, we present findings indicating that the majority of patients are prescribed antimicrobials by a doctor. Two-thirds of the patients wanted information on the etiology of the infection and half of them requested information on optimal management of the infection. All wanted antimicrobial-specific information. Also, about half of the respondents supported doctors using electronic platforms to access medical information during a consultation. In addition, ownership of electronic devices was relatively high within our sample population of people attending NHS outpatient pharmacies. Three-quarters of those who were recently prescribed an antimicrobial stated that they owned a computer and nearly half of respondents owned an Android or Apple mobile phone. Although only 30% of respondents used apps, 95% suggested useful features to include in a bespoke app targeting antimicrobials/infections. We also identified that a significant number of those attending outpatient clinics in a hospital setting, which might also include members of the general public or even NHS staff, besides patients, were not always satisfied with the quality of information received. Overall, our findings suggest that there is a gap and demand by some patients and possibly members of the public for an app that provides antimicrobial- and infection-specific information. Collectively, the global themes that emerged from the respondents’ feedback were knowledge, technology, and patient experience.

### Patient Information Leaflets

Patient information leaflets failed to provide patients with easily understandable and useful information (eg, *“Leaflets included with medicines are a pain,”* *“Too clumsy and contain information overload”*). Some respondents suggested that a dedicated app with practical information might address this gap.

A recent Patient Safety Alert [25] has emphasized the need for health care institutions to engage with patients in order to optimize the use of antimicrobial agents and help prevent the spread of resistant microorganisms. This alert also references the National Institute for Health and Care Excellence (NICE) guidance on antimicrobial stewardship [26], which focuses on patient-centered care and involving patients in their treatment plans, also noting their perceptions about antimicrobial prescribing, whether or not an antimicrobial is indicated.

### Information on Infections/Antimicrobials

Some respondents had scientifically incorrect views and opinions about the misuse of antimicrobials, which clearly indicates the need for targeted, patient-centered antimicrobial stewardship educational interventions. For instance, one patient stated, *“Information needed on gaps one should leave between antibiotic courses, in order to reduce problems with resistance.”* Another patient stated, *“Antibiotics are good, but immune system becomes so depleted after a while. When younger, had tonsillitis and always on antibiotics and effects being experienced in adulthood.”* Echoing the findings highlighted in the Wellcome report [21], our respondents constructed an understanding of antimicrobial resistance around the idea of their bodies *“getting used to”* antibiotics rather than as an adaptive response from microorganisms. As mentioned in the report [21], there is an urgency to address these misconceptions from a public health perspective.

### Technology Awareness

While some respondents strongly supported technological innovations in health care—for instance, one respondent stated, *“Doctors need to keep up to date with technology”* —there may be concerns about privacy and medical data protection, as a small proportion of people in our study (13%) were not happy with doctors accessing electronic resources using a mobile phone/tablet during a consult. These feelings may be explained by wider societal concerns about the misuse of confidential information and data breaches, as well as perceptions of the robustness of security mechanisms and tools.

### Personal Experience on Infections/Antimicrobial Prescribing

Some individuals felt strongly about their own personal/family experiences with infections, which highlights the individual angst and emotive issues experienced by people affected by an infectious disease. Examples include the following: *“Antibiotics given too often to patients and GPs [general practitioners] see it as a quick fix to get rid of the patients. There should be limitations in place to decrease antibiotic prescribing”* and *“I had severe allergic reaction to several antibiotics following brain operation and now being seen by [a specialist].”* This could also possibly be linked to the suboptimal level of satisfaction experienced by some of our respondents when they asked their HCPs about their infections/antimicrobials.

### Limitations

Since only 100 respondents completed the survey and we analyzed 99 (99.0%), our results cannot be widely generalized. However, people from multiple NHS sites participated, which included two distinct geographical locations.

Some individuals also chose not to reply to certain questions and this potentially might have affected our final analysis, but we could only analyze the responses that we obtained. We used closed-ended questions which limited the responses, but we tried to balance this by including an *Other, please specify* category for respondents to provide any additional comments, in addition to an open-ended question. Since part of the recruitment coincided with European Antibiotic Awareness Day (EAAD), it cannot be ignored that this public health awareness campaign, and possibly other related media coverage, might have influenced respondents' feedback.

### Comparison With Prior Work

A US study surveyed adult patients attending an emergency department (ED) [27] and found that from 300 respondents, a total of 71% owned a smartphone—from these, 33% were males—with 95% overall stating that they had apps and 44% stating that they had health-related apps. The median age was 29 years. In our study, only 30% of respondents stated that they had health-related apps and the median age of our respondents was 40 years, so it is possible that age may play a role in determining whether patients regularly access health-related apps. Also, since the patients in the US ED study were younger than the ones in this study, and specifically attended the ED, their disease conditions may have been acute, while in our study the respondents may have had more chronic conditions. Another study [28] targeted patients attending an outpatient psychiatric clinic in Boston, MA, USA. A total of 100 patients were given an uncompensated survey (ie, not offered any incentive e.g. payment, vouchers, etc) to complete and 72% stated that they owned a smartphone. Overall, more than 50% indicated that they would use an app daily for monitoring their mental health condition if this was made available. In this study, patients in the 30-45-year-old age range were more likely to show interest in downloading a mobile phone app to monitor their mental health conditions.

With the increasing availability of health care apps for medical and allied health care professionals, and also as highlighted in a systematic review on health care apps for mobile phones [29], we were interested in exploring patients’ opinions on the use of mobile phones/tablets by medical professionals during a consult. Over half of the respondents stated that they were happy for the doctors to use them, but 13% categorically declared that they were not happy; the majority of these respondents were female, but the numbers were small. Nearly one-third of the respondents selected *depends on the situation*, some adding that if the resource was NHS-approved or if their particular clinical condition was exceptional, they would not object. This finding may indicate a lack of trust in the use of highly sensitive confidential information, with the fear that this might be shared with third parties and/or an expectation that their doctor should know it all.

We also wanted to find out what personal electronic devices were being used by respondents, as this information would enable us to assess how patients obtained information and what electronic devices they used. The majority (62/89, 70%) used a desktop or laptop computer. Almost half of the respondents used an Android or Apple mobile phone. Tablet usage was less, with 17% (15/89) using an Android tablet versus 30% (27/89) using an Apple tablet. Interestingly, very few had Windows-based devices; only 1% (1/89) had a Blackberry mobile phone, but 9% (8/89) stated that they did not use any electronic devices. Market research data shows that mobile device usage has overtaken desktop computer usage since 2014; in the United States, mobile digital media time is higher at 51% for mobile devices versus 42% for desktop or laptop computers [30]. UK data [31] demonstrates that the number of adults who own a mobile phone has risen from 61% (Quarter 1, 2014) to 66% (Quarter 1, 2015); the number of people using their mobile handsets to access the Internet has also increased, from 57% (Quarter 1, 2014) to 61% (Quarter 1, 2015). In addition, 4G mobile subscriptions have dramatically increased from 2.7 million (end of 2013) to 23.6 million (end of 2014).

We explored processes around infection and antimicrobial prescriptions and can report that the majority of patients in our study were prescribed antimicrobials for personal use by a medical practitioner. More than half of our respondents obtained information regarding their infection or antimicrobials directly from their doctor, nurse, or pharmacist. However, when asked about their level of satisfaction, it was interesting to note that only 60% felt completely satisfied by the information they received. This finding may pose the question, ”Do patients really understand the information being given to them by health care professionals?“ as this could lead to suboptimal medication compliance. A Japanese study [32] addressed the issue of medication adherence by developing a smartphone medication self-management system based on interviews with 116 patients with chronic illnesses. Separately, a meta-analysis investigating 13 randomized controlled trials (RCTs) [33] found that electronic medication counseling improved adherence with antiretroviral therapy, as well as virologic response, when compared to control groups not receiving the counseling. However, this was only significant when it was one of multiple components in the intervention, not when it was the sole intervention. Our study does not highlight why patients were not satisfied, so there could be many other reasons, possibly related to HCPs not providing sufficient information, HCPs providing an inappropriate format for the intended patient, and a lack of time to establish understanding/address patients’ questions and concerns.

The three most common features that patients desired in an app included the following: treatment of their symptoms (75%), side-effect profile (71%), and duration of common infections (62%). This finding is very useful and could inform the design of a bespoke app targeted at facilitating the appropriate use of antibiotics by patients. It also identifies a potential gap in current public awareness campaigns around infections/antimicrobials, which needs to be addressed. From our results, it could be inferred that patients, and possibly some members of the general public, would like to have targeted information on self-care and management of infections that includes duration of these infections, as well as important considerations relating to antimicrobials.

The IMS Health report [34] issued by the IMS Institute for Healthcare Informatics, USA, identified 165,000 mHealth apps currently available to the general public, with >90% of these being free of charge. Some of these apps may well be infection/antimicrobial related, however, the report states that most have limited functionalities. Separately, a European Union (June 2015) project on eHealth and well-being [35] published a comprehensive list of eHealth apps receiving funding that failed to include any projects on antimicrobials or infections, indicating a significant missed opportunity.

In addition, both the Wellcome Trust report (2015) [21] and a systematic review [22] on the public`s knowledge and beliefs on AMR suggest that more needs to be done to understand how people perceive the problem of antimicrobial resistance. Also, a careful assessment of the processes and methodologies that are needed to employ and better equip both patients and the public needs to be conducted, so they may grasp the basic concepts of this phenomenon and help curb antimicrobial resistance. Although our survey was conducted in hospital outpatient pharmacies where it is expected that most of the customers would be chronic patients receiving hospital treatment, the pharmacies are easily accessed by visitors and even NHS staff, so these too could have contributed to the survey responses—this data was unavailable as we did not capture it; as well, responses were anonymized. Hence, we postulate that the responses given in our survey could also very likely include members of the public.

### Conclusions

Our findings suggest that the patients and possibly the general public would like easily accessible information about the etiology and management of infections. All of our respondents wanted information relating to the antimicrobials prescribed. We found that ownership of mobile electronic devices was high among respondents to our survey and patients are aware of the availability of health care information using modern electronic resources. We found that a significant proportion of respondents also supported the use of electronic platforms by health care professionals to access medical information.

Furthermore, our results show that there is a potential for developing an evidence-based app for patients and possibly the general public targeting infections and antimicrobials. This app would also help support HCPs to provide the necessary information on antimicrobials/infections, as required by national UK guidance.

However, prior to designing and developing an app, further work should be conducted, ideally by designing a new survey seeking more in-depth information about how the creation of a bespoke app would be used by patients. In addition, focus group interviews with patients and the public should be employed to address some of the misconceptions identified in our study around the generation of antimicrobial resistance, as well as the global themes concerning knowledge, technology, and patient experience around infections and antimicrobial therapy.

## Appendix

Multimedia Appendix 1

## Abbreviations

AMR: antimicrobial resistance
EAAD: European Antibiotic Awareness Day
ECCMID: European Congress for Clinical Microbiology and Infectious Diseases
GP: general practitioner
HCP: health care professional
HPRU: Health Protection Research Unit
NHS: National Health Service
NICE: National Institute for Health and Care Excellence
NIHR: National Institute for Health Research
PHE: Public Health England
RCT: randomized controlled trial
