# Supplementary material for: An Evidence-Based Antimicrobial Stewardship Smartphone App for Hospital Outpatients: Survey-based Needs Assessment Among Patients
Source: JMIR Mhealth Uhealth. 2016 Jul 6;4(3):e83. doi: 10.2196/mhealth.5243 (PMC4954917; doi:10.2196/mhealth.5243)
Supplement: Multimedia Appendix 1 [file mhealth_v4i3e83_app1.pdf]

Antibiotics and mobile device questionnaire
PLUS a chance to win £30 Amazon voucher*

Have you or your child used any antibiotics in the last SIX months or about to start on an antibiotic?**
Are you the carer of someone who is using or used any antibiotics in the last SIX months?**
If your answer is yes to either of the above, then please complete this questionnaire.

This survey is being carried by Imperial College Healthcare NHS Trust and Imperial College. The findings will help the trust develop improvements to the provision of infection and antibiotics information to patients, such as via mobile devices. Participation is voluntary. Your responses will be kept confidential; it will only be used in combination with other participants' responses and will not be identifiable to any individual.

Q1. Who was most recently on an antibiotic in the last six months and when?

- ☐ Me When? ………………………………
- ☐ My child When? ………………………………
- ☐ Someone else for whom I am the carer When? ………………………………

Q2. Who decided that the person needed antibiotics?

- ☐ Hospital doctor
- ☐ Hospital nurse
- ☐ Hospital pharmacist
- ☐ Me, I knew which antibiotic was needed
- ☐ GP
- ☐ Nurse in GP practice
- ☐ Other. Please specify: ……………………………

Q3. What information (if any) did you want to know about the <u>INFECTION / ILLNESS</u> and were you able to get the information? **Please write in the information you would have liked to have.**

	Yes, I got the information	Partially	No, I did not receive the information	No, I did not ask
(i)…………………………………………………………………………	☐	☐	☐	☐
(ii) …………………………………………………………………………	☐	☐	☐	☐
(iii)…………………………………………………………………………	☐	☐	☐	☐

Q4. What information (if any) did you want to know about the <u>ANTIBIOTICS</u> and were you able to get the information?

	Not applicable	Yes, I got the information	Partially	No, I did not receive the information	No, I did not ask
(i) The best time of day to take the antibiotics	☐	☐	☐	☐	☐
(ii) If the antibiotics should be taken with food	☐	☐	☐	☐	☐
(iii) If alcohol can be consumed	☐	☐	☐	☐	☐
(iv) If the antibiotics could be taken at the same time as other medicines	☐	☐	☐	☐	☐
(v) What to do if a dose was missed	☐	☐	☐	☐	☐
(vi) Possible side effects	☐	☐	☐	☐	☐
(vii) Other. Please specify:	☐	☐	☐	☐	☐
………………………………………………………………………	☐	☐	☐	☐	☐

Q5. Where/from whom did you find any of the information in Q3 and Q4, and how satisfied were you? **(please select all that apply)**

	Not applicable	Completely satisfied	Partially satisfied	Not satisfied
(i) Hospital doctor	☐	☐	☐	☐
(ii) Hospital nurse	☐	☐	☐	☐
(iii) Hospital pharmacist	☐	☐	☐	☐
(iv) Family and friends	☐	☐	☐	☐
(v) I searched the internet	☐	☐	☐	☐
(vi) GP	☐	☐	☐	☐
(vii) Other. Please specify: …………………………………………	☐	☐	☐	☐

**Closing date for the prize draw is 26th September 2014. Only eligible participants who are 18 years or over, have fully completed the questionnaire, and provided their contact details will be entered into the prize draw to win the £30 Amazon voucher. The winner will be drawn randomly and contacted via the email address provided by 14th October 2014. **Antibiotic use includes any medicines used for an infection, by any route of administration (including eye drops, tablets, and injections), and obtained by any method (e.g. prescription, internet, over the counter).*

Q6. Which of the following do you have and what do you use it for? ***(Skip to Q9 if you do not have a computer, smartphone or tablet computer).***

		I don't have one	Make/ receive calls	Internet access	Playing games	Download 'apps'	Access medical or health information
(i)	Desktop or laptop computer	☐	☐	☐	☐	☐	☐
(ii)	Android smartphone e.g. *Samsung Galaxy*	☐	☐	☐	☐	☐	☐
(iii)	Android tablet e.g. *Kindle Fire*	☐	☐	☐	☐	☐	☐
(iv)	Apple smartphone e.g. *iPhone*	☐	☐	☐	☐	☐	☐
(v)	Apple tablet e.g. *iPad*	☐	☐	☐	☐	☐	☐
(vi)	Windows-based smartphone e.g.*Nokia Lumia*	☐	☐	☐	☐	☐	☐
(vii)	Windows-based tablet e.g. *Microsoft Surface*	☐	☐	☐	☐	☐	☐
(viii)	Blackberry smartphone	☐	☐	☐	☐	☐	☐
(ix)	Other computing device. Please specify: …………………………………………………………………	☐	☐	☐	☐	☐	☐

Q7. Do you use any apps about health, health care, medicines or illnesses on your mobile device (smartphone or tablet computer)?

☐ Yes ☐ No

If yes, can you tell us the name of the apps?……………………………………………………………………………………

Q8. Thinking about infections and antibiotics, for which of the following would you use an app? **(please select all that apply)**

☐ Find information about usual length of illness caused by common infections
☐ Find information about how I can treat my symptoms
☐ Find information about whether or not antibiotics are needed
☐ Find out about the side effects of antibiotics
☐ Find out if I need to see my doctor
☐ Find out about the signs of bacterial versus viral chest infections
☐ Find tips on how to reduce the risk of getting common infections
☐ Recording of antibiotic treatment information by you or your doctor
☐ Other. Please describe:……………………………………………………………………………………………

Q9. How would you feel if a doctor used a smartphone or tablet computer to find healthcare information in front of you?

☐ Fine, no problem
☐ Not fine, they should not be using it
☐ Depends on the situation
☐ Other. Please describe: ……………………………………………………………………………………………

Q10. Please provide any further feedback that you would like to give about the topic of the survey e.g. recent antibiotic experience, or about the survey itself:

Q11. (a) About you. Are you: Male ☐ Female ☐

(b) What is your age? ……………………

(c) Education: ☐ Degree, or Degree equivalent and above
☐ Other qualifications
☐ No qualifications

Q12. Would you be interested in any of the following?

☐ I would like to receive a summary of the results
☐ I would like to be invited for future research in this area

Please provide your contact details if you selected one or both of the above in Q12.

Name: ……………………………………………………… Email: ………………………………………………………

Please provide your contact details if you would like to be entered into the prize draw:

Name: ……………………………………………………… Email: ………………………………………………………

Thank you for your time.
Please return the questionnaire to the hospital pharmacy staff or place in the **'Antibiotics and mobile device questionnaire' box at the pharmacy reception.** For more information, email pocast@imperial.nhs.uk

**Closing date for the prize draw is 26th September 2014. Only eligible participants who are 18 years or over, have fully completed the questionnaire, and provided their contact details will be entered into the prize draw to win the £30 Amazon voucher. The winner will be drawn randomly and contacted via the email address provided by 14th October 2014. **Antibiotic use includes any medicines used for an infection, by any route of administration (including eye drops, tablets, and injections), and obtained by any method (e.g. prescription, internet, over the counter).*
